# Total endoscopic left ventricle lipoma removal

**DOI:** 10.1186/s13019-021-01602-y

**Published:** 2021-08-04

**Authors:** Tom Langenaeken, Aydin Basoglu, Abdullah Kaya, Alaaddin Yilmaz

**Affiliations:** 1 Department of Cardiac Surgery, JESSA Hospitals Hasselt, Stadsomvaart 11, 3500 Hasselt, Belgium; 2 Department of Cardiology, MOL Hospitals, Mol, Belgium

**Keywords:** Cardiac lipoma, Radical resection, Minimally invasive, Clinical management, Case report

## Abstract

**Background:**

Left ventricle (LV) lipoma is a very rare, benign cardiac tumor. Due to its rarity, LV lipoma is often misdiagnosed. Aspecific symptoms such as murmurs, arrhythmias, memory loss and palpitation may occur due to the mass effect.

**Case presentation:**

We report a case report of a 42 year old woman who was found to have left ventricle mass after check-up for arrhytmia. By a fully endoscopic approach, the mass was successfully resected from the left ventricle without the need for sternotomy.

**Conclusion:**

Total endoscopic removal of left ventricle lipoma’s can be done safely and has several advantages to conventional sternotomy. Larger studies are needed to confirm this hypothesis.

## Introduction

Cardiac lipoma is a rare primary cardiac tumor. Origin in the left ventricle is seen in 33% of cases, with 57% of these cases the primary site being intracavity [1]. Almost two thirds of patients become symptomatic due to the mass effect. A broad variety of symptoms from chest pain to memory loss has been reported, though dyspnea is the most common. Diagnosis is made by ultrasonography supplemented by computed tomography (CT) or magnetic resonance imaging (MRI). Radical resection is indicated in all cases of cardiac lipomas even when asymptomatic, due to the risk of overgrowth and infiltration in adjacent structures. Surgical resection is usually done by either full or partial resection. We propose a minimally invasive, non-sternotomy approach.

## Case presentation

**Fig. 1:**
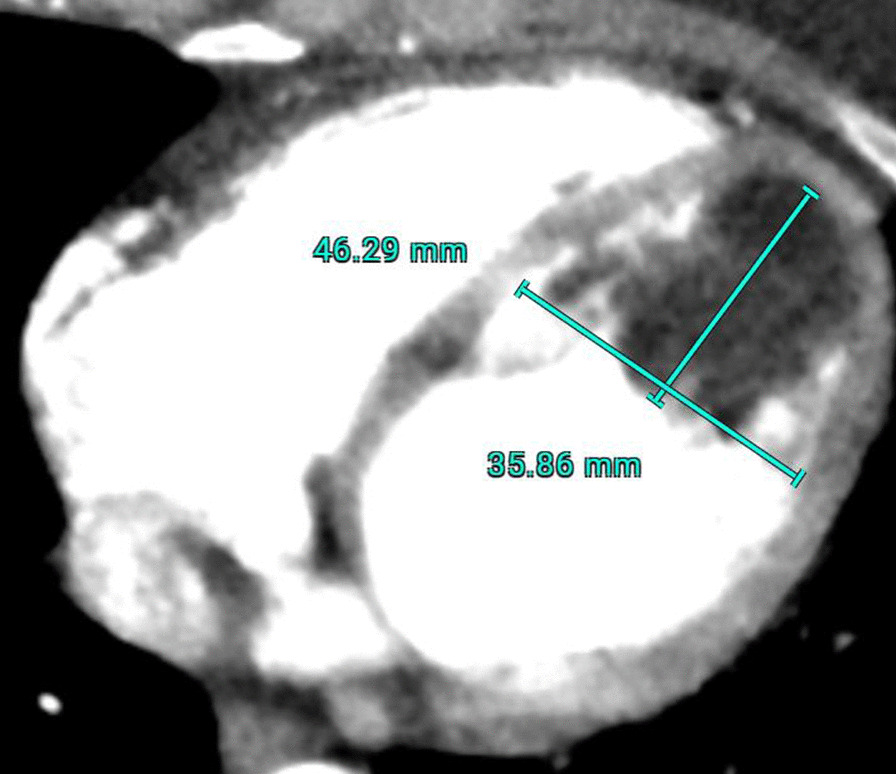
CT image of the LV lipoma

A 42-year-old woman was referred to our hospital due to detection of supraventricular arrhythmia during a dental procedure under general anesthesia. She was asymptomatic besides sporadic memory loss. Medical history was insignificant. Physical examination showed a body mass index (BMI) of 24 kg/cm^2^, normal pulse of 80 bpm, blood pressure 130/90 mmHg. She had no pathological murmurs nor any other abnormal findings. 12-lead electrocardiography (ECG) and exercise test were normal. Further evaluation by transesophageal ultrasound (US) and CT showed a globular mass attached to the apex of the left ventricle measuring 4.6 × 3.6 cm (Fig. 1). No other abnormalities were found and echinococcus-serology was negative. Decision for minimally invasive resection was made.

**Fig. 2:**
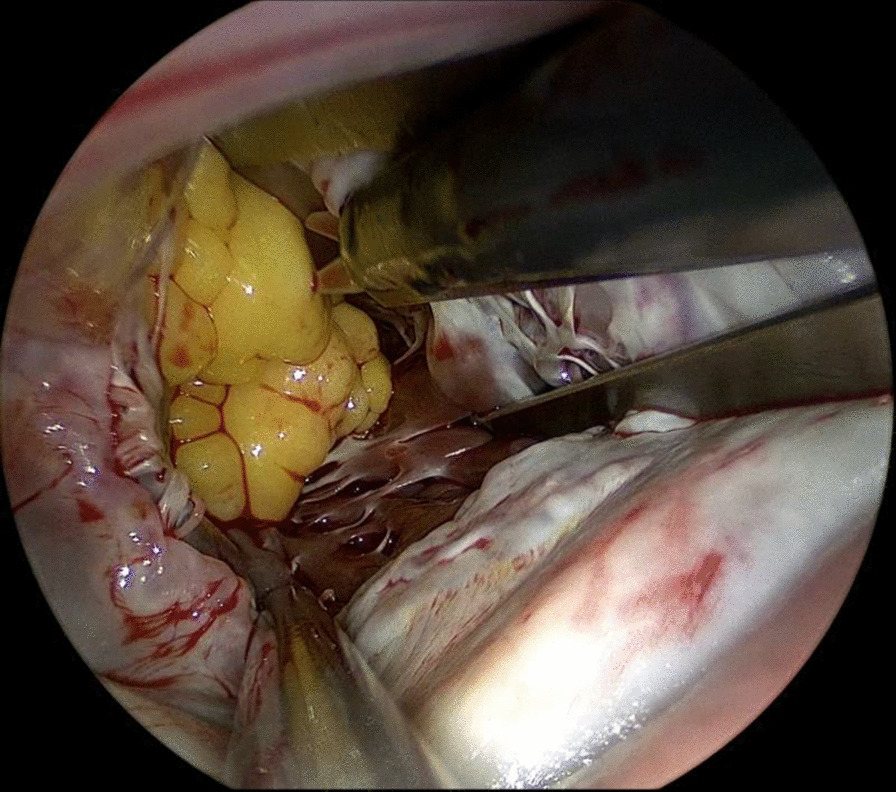
Thoracoscopic view of the LV lipoma

**Fig. 3:**
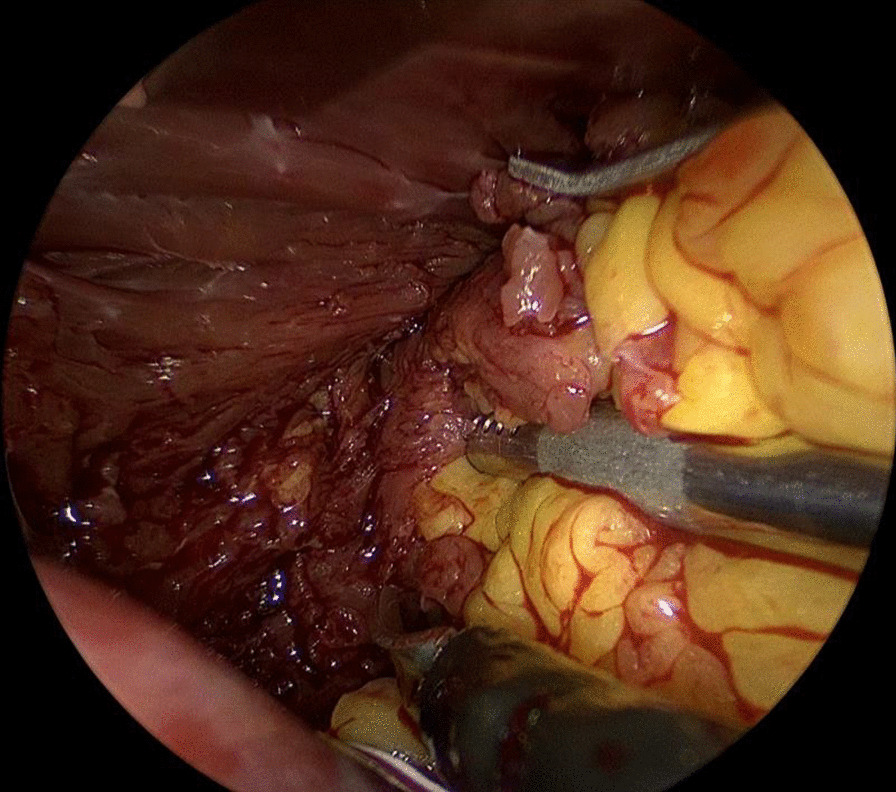
Dissection of the LV lipoma from the LV wall

**Fig. 4:**
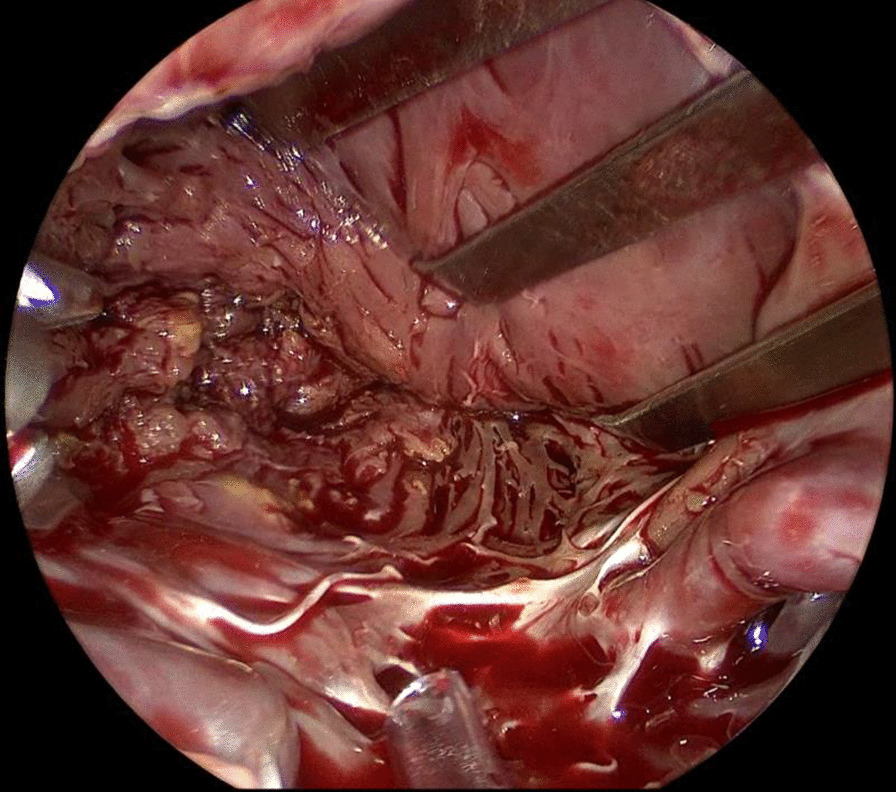
Inspection of the LV wall after resection

After general anesthesia and standard draping, connection to the extracorporeal circulation (ECC) was made by femoral cannulation combined with jugular venous return. 3 trocars were placed in second to fourth intercostal space in the right axillary line. 1.5 cm utility port was placed in the third intercostal space. 5 mm thoracoscope was used for vision. Pericardium was opened followed by aortic cross clamping and anterograde cardioplegia was given. Left atrium was accessed via Waterston’s groove. Clearly visible behind the mitral valve is a lobulated, fatty mass with a broad base pedunculated to the apex of the left ventricle (Fig. 2). The mass has partial ingrowth in the left ventricular wall, but can be resected at a macroscopic safe margin using endoscopic scissors and forceps (Fig. 3). Attention was given to leaving enough ventricular wall intact for safe ventricular function. Inspection showed no residual lipoma tissue (Fig. 4). Left atrium was closed by continuous suture. Pericardium was closed and one thorax drain was left in the right pleural cavity. Patient was disconnected from the ECC and incisions were closed in layers.

Postoperative stay was uneventful and after 7 days patient was discharged. Histological examination was consistent with lipoma. Final measurements were 5.5 × 3.5 × 3.5 cm. Further staining for liposarcoma was negative. Mouse double minute 2 homolog (MDM2) fluorescence in situ hybridization (FISH) was negative for malignant lipomatous tumor. Echocardiogram showed no signs of recurrence during a 2-month follow up period.

## Discussion

Up to date, no non-sternotomy minimally invasive approach has been reported. The technique described in this report allows adequate access to the left ventricle via the left atrium through the mitral valve by use of a utility port (1.5 cm) and 3 trocars entry points. Thorough inspection of the left ventricle is possible allowing for meticulous dissection of the LV lipoma from the left ventricular wall while keeping the lipoma itself intact, minimising the risk of embolization. Lipoma growth is from interior to exterior, hence an approach from inside the ventricle allows good differentiation between LV wall and lipoma. Sternotomy comorbidity is avoided allowing for lesser postoperative pain, faster mobilisation and recovery.

Literature about these rare cardiac tumors is mostly limited to isolated case reports, recently collected in a large systematic review [1]. Of all primary tumors, 0.2 to 0.4% are from cardiac origin. From these, only 8.4% are lipoma’s mostly presenting in the 40–70 year old age group with no gender predilection. Growth is slowly and asymptomatic in its early stages. Up to 57% of patients become symptomatic due to the mass effect on adjacent structures [1]. Echocardiogram is the preferred diagnostic tool LV lipomas: tumor shape and size, adjacent structures and interference with in- and outflow tracts can be evaluated. In addition, CT or MRI can further objectivate tumor density, blood supply and (extra)cardiac invasion. Postoperative histological examination is crucial for diagnosis confirmation. MDM2 FISH test is needed in adjunct to histology to distinguish between lipoma and atypical lipomatous tumor [2].

Despite no official guideline for treatment of lipoma, current practice is surgical resection by either partial or full sternotomy. Some have reported thoracoscopic techniques, though sternotomy was still needed [3, 4]. Other approaches such as by ventriculotomy have also been described, though we deem this approach high risk due to weakening of the ventricular wall and possible lesion of the coronary arteries [5].

## Conclusion

LV lipoma is a very rare, benign cardiac tumor. Once patients become symptomatic, resection should be considered. Standard surgical approach is by sternotomy. We think a fully endoscopic approach is feasible and beneficial for most patients.

## Data Availability

All data is available.

## Abbreviations

LV: Left ventricle
CT: Computed tomography
MRI: Magnetic resonance imaging
BMI: Body mass index
ECG: Electrocardiography
US: Ultrasound
MDM2: Mouse double minute 2 homolog
FISH: Fluorescence in situ hybridization
